# Prescriber's Companion

**Published:** 1892-06

**Authors:** 


*Prescriber's Companion.* By THOMAS SAVILL, M.D., M.R.C.P. 2nd Edition. Pp. 48. London: JOHN BALE & SONS. [n.d.]

This is greatly enlarged from the first edition; it is still small but contains a large amount of valuable information on medicinal and general therapeutics in a small space, and is one of the best of the little books of this kind, which, judging from the supply, meet a wide-spread demand.

